# Nutritional screening based on objective indices at admission predicts in-hospital mortality in patients with COVID-19

**DOI:** 10.1186/s12937-021-00702-8

**Published:** 2021-05-25

**Authors:** Feier Song, Huan Ma, Shouhong Wang, Tiehe Qin, Qing Xu, Huiqing Yuan, Fei Li, Zhonghua Wang, Youwan Liao, Xiaoping Tan, Xiuchan Song, Qing Zhang, Daozheng Huang

**Affiliations:** 1 Department of Emergency and Critical Care Medicine, Guangdong Provincial People’s Hospital, Guangdong Academy of Medical Sciences, Guangzhou, 510080 China; 2 Department of Cardiology, Guangdong Provincial People’s Hospital, Guangdong Academy of Medical Sciences, Guangdong Provincial Cardiovascular Institute, Guangzhou, 510080 China; 3 Department of Critical Care Medicine, Guangdong Provincial People’s Hospital, Guangdong Academy of Medical Sciences, Guangdong Provincial Geriatrics Institute, Guangzhou, 510080 China; 4 Department of Emergency Medicine, Shanghai Jiao Tong University Affiliated Sixth People’s Hospital, Shanghai, 200233 China; 5 Department of Respiratory and Critical Care Medicine, the First People’s Hospital of Shaoguan, Shaoguan, 512000 China; 6 Department of Emergency, the First Affiliated Hospital of Jingzhou, Jingzhou, 434000 China; 7 Department of Gastroenterology, the First Affiliated Hospital of Yangtze University, Jingzhou, 434000 China; 8 Department of Critical Care Medicine, Dongguan Eighth People’s Hospital, Dongguan Children’s Hospital, Dongguan, 523000 China

**Keywords:** Mortality, COVID-19, Malnutrition, Coronavirus, Nutrition

## Abstract

**Background:**

Could nutritional status serve as prognostic factors for coronavirus disease 2019 (COVID-19)? The present study evaluated the clinical and nutritional characteristics of COVID-19 patients and explored the relationship between risk for malnutrition at admission and in-hospital mortality.

**Methods:**

A retrospective, observational study was conducted in two hospitals in Hubei, China. Confirmed cases of COVID-19 were typed as mild/moderate, severe, or critically ill. Clinical data and in-hospital death were collected. The risk for malnutrition was assessed using the geriatric nutritional risk index (GNRI), the prognostic nutritional index (PNI), and the Controlling Nutritional Status (CONUT) via objective parameters at admission.

**Results:**

Two hundred ninety-five patients were enrolled, including 66 severe patients and 41 critically ill patients. Twenty-five deaths were observed, making 8.47% in the whole population and 37.88% in the critically ill subgroup. Patients had significant differences in nutrition-related parameters and inflammatory biomarkers among three types of disease severity. Patients with lower GNRI and PNI, as well as higher CONUT scores, had a higher risk of in-hospital mortality. The receiver operating characteristic curves demonstrated the good prognostic implication of GNRI and CONUT score. The multivariate logistic regression showed that baseline nutritional status, assessed by GNRI, PNI, or CONUT score, was a prognostic indicator for in-hospital mortality.

**Conclusions:**

Despite variant screening tools, poor nutritional status was associated with in-hospital death in patients infected with COVID-19. This study highlighted the importance of nutritional screening at admission and the new insight of nutritional monitoring or therapy.

## Introduction

On 11 March 2020, the World Health Organization (WHO) declared the pandemic outbreak of coronavirus disease 2019 (COVID-19). Globally, as of 20 August 2020, there have been 22,213,869 confirmed cases of COVID-19, including 781,677 deaths, reported to WHO [1]. The severity of COVID-19 ranges from mild to critical illness. Elderly patients and those with comorbidities (such as diabetes, cardiovascular disease, and chronic respiratory disease) are the most vulnerable to death and at risk of malnutrition/undernutrition [2–5]. Previous studies expressed concerns for severe pneumonia patients, who encountered protein loss, resulting in impairing the immune defense system [6]. The COVID-19 patients also had signs of protein loss such as reduced levels of albumin and impaired organ function [7], highlighting the role of nutrition risk screening and its prognostic value for COVID-19 patients [8]. Nutritional screening should be simple and non-invasive. The Controlling Nutritional Status (CONUT) score is a practical tool that helps evaluate protein reserves, calorie expenditure, and immune defenses, based on serum albumin, total cholesterol and lymphocytes count [9]. The prognostic nutritional index (PNI), calculated by serum albumin level and lymphocyte count, reflects the immunological nutritional condition [10]. The geriatric nutritional risk index (GNRI) was developed as a simplified screening tool based on serum albumin and body mass index (BMI) [11]. However, evidence regarding the abovementioned nutritional screening tools and their association with clinical outcomes for COVID-19 patients remained unclear. The objective of the present study aimed to investigate whether nutritional status at admission predicted in-hospital mortality in patients with COVID-19.

## Method

### Study design

The present study was a retrospective, observational study conducted at Leishenshan Hospital and the First People’s Hospital of Jingzhou, China. Consecutive cases, diagnosed by the nucleic acid-positive test and typed as mild, moderate, severe, or critically ill (following the diagnosis protocol for COVID-19 [12]), were enrolled between January 2020 and May 2020. The exclusion criteria were 1) pregnancy or 2) less than 18 years old. The data were anonymous, and the *informed consent was waived* for the retrospective, observational design, which *was* approved by the Ethics Committee of the First People’s Hospital of Jingzhou and Guangdong Provincial People’s Hospital.

### Data collection

Demographic, medical history, laboratory tests, chest computed tomography (CT) findings, and in-hospital mortality were recorded. Bodyweight and height were measured at admission or self-reported by patients. Blood sampling and CT examinations were performed within 12 h of admission. Comorbidity was ascertained by the patients’ self-report of history, physician-documented history, or diagnosis during hospitalization. The severity of chest CT findings was based on the baseline chest CT features. Following the diagnosis protocol [12], it was defined as normal, ground-glass opacities (GGO), focal GGO with or without consolidation, bilateral involvement, diffuse and interstitial lesions.

### Assessment of nutrition status

Baseline GNRI was calculated from serum albumin values and body weight and height obtained on hospital admission as follows:

GNRI = 14.89 × serum albumin (g/dL) + 41.7 × (measured body weight (kg)/ideal body weight (kg)).

The ideal body weight was defined as the value calculated using the Lorentz-formula.

Ideal body weight = (height (cm) − 100) − (height (cm) − 150) / 4 for men and (height (cm) − 100) − (height (cm) − 150) / 2 for women [11].

In the previous study, GNRI cutoff values were calculated by using albumin and weight loss in the elderly, which were defined 4 grades of nutrition-related risk, including major, moderate, low, and no risk. The risk escalated accompanied by the decrease of GNRI [11]. However, the present study focused on the patients with COVID-19 instead of merely the elderly, so the grading was not implemented in the present study.

Baseline CONUT score was calculated from serum albumin levels, total cholesterol levels and total lymphocyte counts as previously reported [9]. Patients with CONUT scores of 0–1 have a normal nutritional status, those with CONUT scores of 2–4 have a light degree of undernutrition, those with CONUT scores of 5–8 have a moderate degree of undernutrition, and those with CONUT scores of 9–12 have a severe degree of undernutrition (Table 1).

**Table 1:** Assessment of undernutrition degree by controlling nutritional status (CONUT) score

Parameter	Score			
Serum albumin, g/L (score)	≥35.0 (0)	30.0–34.9 (2)	25.0–29.9 (4)	<25.0 (6)
Total lymphocyte count, X10^9^/L (score)	≥1.60 (0)	1.20–1.59 (1)	0.80–1.19 (2)	< 0.80 (3)
Total cholesterol, mg/dl (score)	≥180 (0)	140–179 (1)	100–139 (2)	< 100 (3)
Dysnutritional state (total score)	Normal (0–1)	Light (2–4)	Moderate (5–8)	Severe (9–12)

The PNI was calculated by a formula as follows:

PNI score: 10 × serum albumin (g/dL) + 0.005 × total lymphocyte count (mm3). Patients with a PNI > 38, PNI of 35–38, PNI < 35, reflected normal, at moderate, and at severe risk of malnutrition, respectively [10].

### Statistical analysis

Continuous variables were expressed as the means ± standard deviation (SD) or the medians (interquartile range, IQR). Categorical variables were expressed as numbers (percentages). Patients were classified into 3 groups (mild or moderate, severe, and critically ill) according to the severity of COVID-19. The differences in the demographic data and laboratory variables were assessed across 3 groups. Continuous variables were compared using the one-way analysis of variance (ANOVA) or the Kruskal-Wallis test. Categorical variables were compared using Fisher’s exact test or the Chi-squared test. Instead of adopting the established grading of these nutritional risk scores, the present study attempted to recalculate the most appropriate cut-off value in the COVID-19 population, using the receiver operating characteristics curve. Logistic regression models were used to determine the association between risk for malnutrition (treated as either a continuous variable or a categorical variable) and in-hospital death after adjusted the covariates. Variables used in the models of multivariate analysis were selected under the previous studies [13, 14]. Levels of GNRI, PNI, and CONUT were included in multivariate modeling respectively, and odds ratios (ORs) and 95% confidence intervals (CIs) were calculated. To assess the accuracy of predicting in-hospital mortality, the C-index was calculated. A *p*-value < 0.05 was considered statistically significant. Calculations were carried out using R version 4.0.2 (http://www.R-project.org/; R Foundation for Statistical Computing, Vienna, Austria).

## Results

### Population characteristics

Two hundred ninety-five patients were enrolled in the final analysis, including 5 mild patients, 183 moderate patients, 66 severe patients, and 41 critically ill patients. They were divided into 3 groups: (1) mild or moderate (*n* = 188), (2) severe (*n* = 66), and (3) critically ill (*n* = 41). Table 2 showed the baseline clinical characteristics of all patients, and the patients were divided according to the severity of COVID-19. The median age of the included patients was 58.00 (IQR: 44.00–69.00) years. The age of participants in the mild/moderate group (51.50 years, IQR: 38.00–64.00) was significantly younger than the severe group (67.00 years, IQR: 57.25–76.75) and the critical ill group (66.00 years, IQR: 61.00–75.00) (*p*<0.0001). Male patients accounted for 52.5% (*n* = 155) of the study population. The proportions of males were 46.81% in the mild/moderate group, 56.06% in the severe group, and 73.17% in the critical ill group (*p* = 0.0074). The most common comorbidities were cardiovascular diseases (34.24%), diabetes (24.07%), chronic liver disease (8.14%), and chronic respiratory disease (7.80%). The prevalence of cardiovascular diseases went significantly higher along with the exacerbation of COVID-19. The prevalence of chronic kidney disease was significantly higher in critically ill patients. The proportions of comorbid chronic respiratory disease were 5.32% in the mild/moderate group, 15.15% in the severe group, and 7.32% in the critical ill group (*p* = 0.0372). Other characteristics and had no significant difference among the 3 groups.

**Table 2:** Baseline characteristics of the patients among different severity of COVID-19

	All cases *N* = 295	Mild/moderate *N* = 188	Severe *N* = 66	Critical ill *N* = 41	*p*-value
Age, years	58.00 (44.00–69.00)	51.50 (38.00–64.00)	67.00 (57.25–76.75)	66.00 (61.00–75.00)	< 0.0001
Male, n, %	155,52.54%	88,46.81%	37,56.06%	30,73.17%	0.0074
Smoking, n, %	17,5.76%	7,3.72%	5,7.58%	5,12.20%	0.0836
Alcohol history, n, %	16,5.42%	7,3.72%	4,6.06%	5,12.20%	0.0918
Diabetes mellitus, n, %	71,24.07%	39,20.74%	17,25.76%	15,36.59%	0.0928
Cardiovascular disease, n, %	101,34.24%	49,26.06%	26,39.39%	26,63.41%	< 0.0001
Chronic respiratory disease, n, %	23, 7.80%	10, 5.32%	10,15.15%	3, 7.32%	0.0372
Chronic liver disease, n, %	24,8.14%	14,7.45%	4,6.06%	6,14.63%	0.2446
Chronic kidney disease, n, %	18,6.10%	7,3.72%	3,4.55%	8,19.51%	0.0006
Chest CT features, n, %					< 0.0001
Normal	2, 0.70%	2, 1.08%	0, 0.00%	0, 0.00%	
GGO	111, 38.68%	81, 43.78%	26, 41.94%	4,10.00%	
Focal GGO +/− consolidation	18, 6.27%	18, 9.73%	0, 0.00%	0, 0.00%	
Bilateral involvement	134, 46.69%	79, 42.70%	30, 48.39%	25,62.50%	
Diffuse and interstitial lesions	22, 7.67%	5, 2.70.00%	6,9.68.00%	11,27.50%	

Continuous variables were expressed as the means ± standard deviation or the medians (interquartile range) in accordance with the distribution. The differences in the baseline variables were assessed across the mild/moderate, severe, and critical ill groups. Continuous variables were compared using the one-way analysis of variance or the Kruskal-Wallis test. Categorical variables were compared using Fisher’s exact test or the Chi-squared test. *CT* Computed tomography, *GGO* Ground glass opacities

### Laboratory measurements

Laboratory characteristics were present in Table 3. Overall, conditions of highest body temperature and SaO2 at admission were worse in the patients of the more severe group. The median weight and average BMI were 64 kg (IQR: 58 to 72.5 kg) and 24.06 ± 3.18 kg/m2, respectively. On admission, lymphocyte counts, platelet counts, and hematocrit were lower in severe and critically ill patients than in mild/moderate patients (*p* < 0.0001, *p* = 0.0389, and *p* = 0.0061 respectively). D-dimer on admission was higher along with the severity of the disease (*p* < 0.0001). Most patients, especially critically ill patients, had significant changes in inflammatory markers, C-reactive protein (CRP) and interleukin 6 (IL-6) levels presented a similar increasing trend in terms of the severity. For nutrition-related indicators, the albumin levels were significantly lower in critically ill patients than those of severe or mild/moderate patients (*p* < 0.0001). On the contrary, the levels of blood urea nitrogen and serum creatinine were elevated in critically ill patients (*p* < 0.0001 and *p* = 0.0002, respectively). However, there was no difference in initial levels of weight, body mass index, or lipid parameters including total cholesterol and triglycerides values.

**Table 3:** Baseline laboratory measurements of patients among different severity of COVID-19

	All cases *N* = 295	Mild/moderate *N* = 188	Severe *N* = 66	Critical ill *N* = 41	*p*-value
Weight, kg	64.00 (58.00–72.50)	63.00 (57.00–73.00)	65.00 (60.00–72.00)	66.00 (60.00–68.00)	0.6640
Body mass index, kg/cm^2^	24.06 ± 3.18	24.48 ± 3.75	23.65 ± 1.95	22.70 ± 1.82	0.1320
Index body temperature, °C	36.70 (36.40–37.10)	36.70 (36.40–37.10)	36.60 (36.30–37.30)	36.70 (36.40–37.10)	0.9210
Highest body temperature, °C	37.60 (37.00–38.40)	37.40 (37.00–38.00)	37.80 (37.20–38.40)	38.60 (38.00–39.30)	< 0.0001
Index SaO2, %	98.00 (97.50–99.00)	99.00 (98.00–99.00)	98.00 (96.00–99.00)	97.50 (90.50–98.75)	0.0019
Lowest SaO2, %	96.00 (94.00–97.00)	96.00 (95.00–98.00)	95.00 (92.00–96.00)	85.00 (71.00–92.00)	< 0.0001
Alanine transaminase, U/L	29.00 (15.00–58.00)	26.50 (14.75–51.00)	38.00 (17.00–107.50)	37.00 (15.00–64.00)	0.0444
Aspartate transaminase, U/L	27.00 (19.00–44.00)	25.00 (18.00–35.00)	39.00 (20.75–65.25)	30.50 (20.75–58.00)	0.0014
Serum albumin, g/L	40.00 (36.35–42.80)	40.70 (37.28–44.10)	38.20 (35.15–41.63)	36.40 (29.40–40.30)	< 0.0001
White blood cell, ×10^9^/L	6.98 (5.42–10.50)	6.45 (5.29–8.67)	8.00 (5.54–14.00)	11.47 (7.15–15.98)	< 0.0001
Total lymphocyte count, × 10^9^/L	1.63 (1.23–2.03)	1.71 (1.51–2.13)	1.18 (1.60–1.90)	0.64 (0.84–1.30)	< 0.0001
Total platelet count, X10^9^/L	242.00 (180.00–294.00)	243.00 (187.25–290.75)	244.00 (196.25–299.00)	189.00 (125.00–294.00)	0.0389
Hematocrit, %	38.00 (34.30–41.90)	38.10 (34.80–42.50)	37.55 (33.68–41.15)	30.10 (34.45–39.23)	0.0061
D-Dimer, mg/L	0.72 (0.30–2.82)	0.49 (0.23–0.98)	1.81 (0.60–5.23)	6.25 (2.07–10.94)	< 0.0001
C-reactive protein, mg/L	8.94 (1.41–38.69)	4.89 (0.89–19.67)	16.74 (1.34–47.30)	69.53 (21.07–128.89)	< 0.0001
Interleukin-6, pg/ml	3.09 (1.50–18.38)	1.50 (1.50–4.70)	3.89 (1.87–9.09)	60.75 (24.19–213.05)	< 0.0001
Blood Urea Nitrogen, mmol/L	4.91 (3.70–6.78)	4.35 (3.37–5.86)	5.48 (4.19–7.00)	9.56 (5.91–13.40)	< 0.0001
Serum creatinine, μmol/L	67.10 (55.30–83.00)	63.40 (52.50–76.80)	71.70 (59.93–85.25)	82.20 (63.50–142.78)	0.0002
Total bilirubin, μmol/L	10.15 (7.40–13.45)	10.10 (7.53–13.38)	9.85 (7.45–12.43)	11.85 (7.10–16.43)	0.2957
Total cholesterol, mmol/L	3.99 ± 1.08	4.06 ± 1.06	4.10 ± 1.01	3.72 ± 1.21	0.3470
Triglyceride, mmol/L	1.19 (0.82–2.01)	1.24 (0.85–2.19)	1.08 (0.74–1.88)	1.01 (0.85–1.99)	0.4634

Continuous variables were expressed as the means ± standard deviation or the medians (interquartile range) in accordance with the distribution. SaO2, oxygen saturation

### Risk for malnutrition

The screening of risk for malnutrition was displayed in Table 4. Most patients, especially critically ill patients, had significant changes in nutrition-related parameters. In the severe and critically ill groups, the PNI scores were more severe than in the mild/moderate group (*p*<0.0001). For the mild/moderate group, 167 (94.89%) patients had normal nutritional statuses on admission, while 59 (90.77%) and only 24 (60.00%) patients in the severe and critically ill group, respectively. For the overall population, the median CONUT scores were 2.50 (IQR: 1.00–5.00). The more severe patients had significantly higher CONUT scores (*p*<0.0001). 75 (67.00%) had light-to-severe nutritional disturbances (light, 36.61%; moderate, 20.54%; severe, 9.82%). For the critically ill group, 24 (60.00%) patients had normal nutritional statuses on admission using PNI score. Meanwhile, no patients were evaluated as normal nutritional status using the CONUT score. The mean GNRI values were 103.82 ± 11.00. The GNRI values decreased as the disease deteriorated (*p* = 0.0018).

**Table 4:** The risk of malnutrition of patients among different severity of COVID-19

	All cases *N* = 295	Mild/moderate *N* = 188	Severe *N* = 66	Critical ill *N* = 41	*p*-value
PNI	48.55 (43.70–52.70)	49.60 (46.49–54.15)	47.20 (41.65–50.70)	40.95 (34.26–46.86)	<0.0001
Malnutrition risk assessed via PNI, n, %					<0.0001
Normal	250,88.97%	167,94.89%	59,90.77%	24,60.00%	
Moderate	13, 4.63%	6, 3.41%	3, 4.62%	4,10.00%	
Severe	18, 6.41%	3, 1.70%	3, 4.62%	12, 30.00%	
CONUT score	2.50 (1.00–5.00)	2.00 (1.00–3.50)	3.00 (1.00–4.50)	5.50 (4.00–9.75)	<0.0001
Malnutrition risk assessed via CONUT score, n, %					<0.0001
Normal	37, 33.04%	27, 45.76%	10, 37.04%	0	
Light	41, 36.61%	22, 37.29%	10, 37.04%	9, 34.62%	
Moderate	23, 20.54%	7, 11.86%	7, 25.93%	9, 34.62%	
Severe	11, 9.82%	3, 5.08%	0, 0.00%	8, 30.77%	
GNRI score	103.82 ± 11.00	106.40 ± 11.30	101.12 ± 8.93	96.01 ± 10.57	0.0018

Continuous variables were expressed as the means ± standard deviation or the medians (interquartile range) in accordance with the distribution. *PNI* Prognostic nutritional index, *CONUT* Controlling nutritional status, *GNRI* Geriatric nutritional risk index

### Receiver operating characteristic curves

**Fig. 1:**
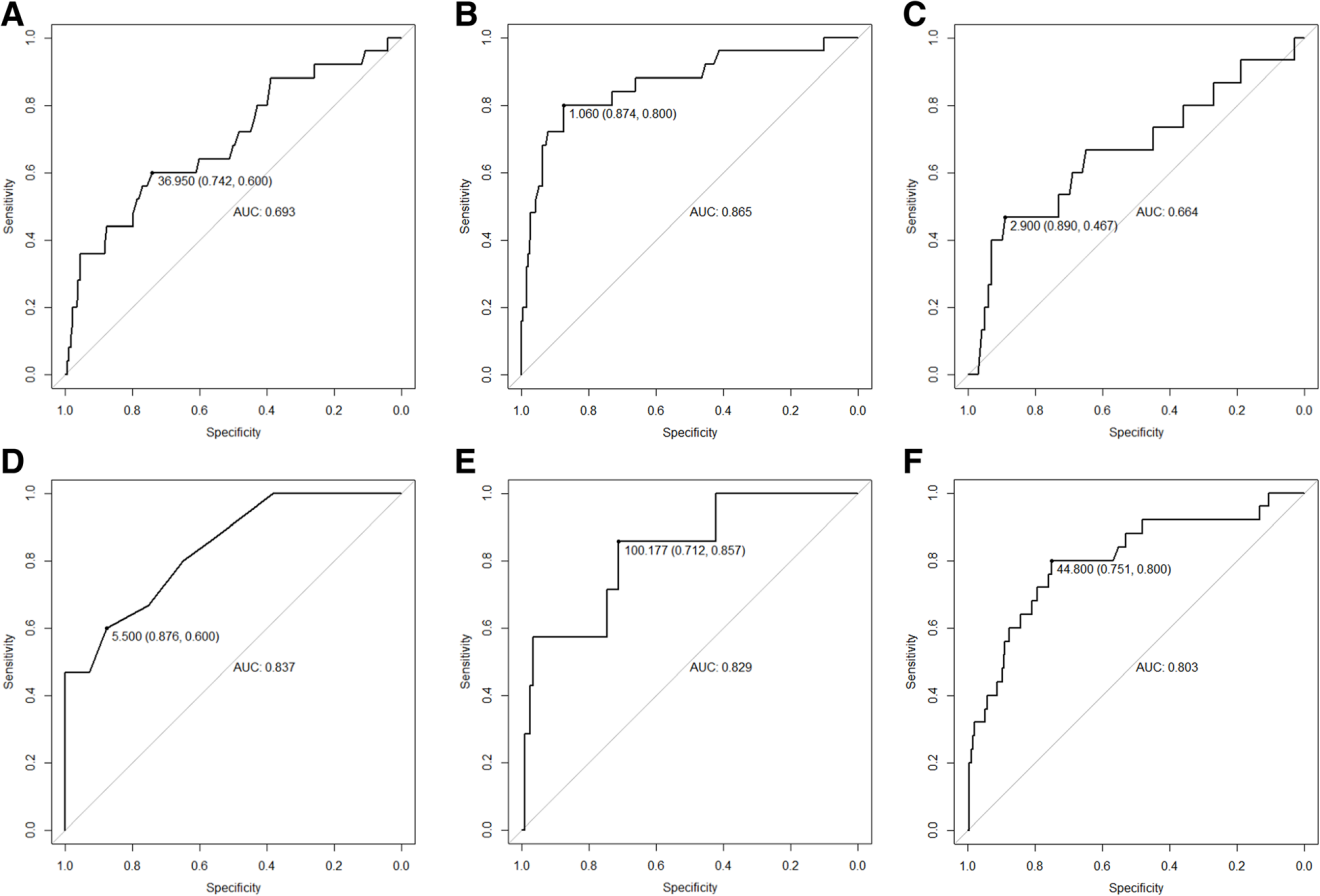
The receiver operating characteristic curves of serum albumin (**a**), total lymphocyte count (**b**), total cholesterol (**c**), controlling nutritional status score (**d**), geriatric nutritional risk index (**e**) and prognostic nutritional index (**f**) for predicting in-hospital mortality

Twenty-five deaths were observed and all of these were critically ill patients. The mortality was 8.47% in the whole population and 37.88% in critically ill patients. Areas of the receiver operating characteristic (ROC) curves were measured for serum albumin, lymphocyte count, total cholesterol, GNRI, CONUT, and PNI, and it confirmed a better prognostic implication of GNRI and CONUT compared to other parameters (Fig. 1). The cut-off value for the CONUT score, GNRI, and PNI were 5.5, 100, and 44.8, respectively.

### Multivariate analysis to evaluate the prognostic implication of nutritional risk score for predicting in-hospital death

Unadjusted univariate and adjusted multivariate logistic regression analysis by multiple models were used to determine the prognostic implications of different nutritional risk scores for in-hospital mortality. The traditional models were shown in Table 5. Table 6 presented the univariate and multivariate associations between CONUT score and in-hospital deaths. The logistic regression analyses revealed that a per point increase in the CONUT score was associated with an increased risk of in-hospital death (OR and 95% CI: 1.58 and 1.28–1.94, *p*<0.0001). The analysis also revealed that COVID-19 patients with worse nutritional disturbances had a higher risk for in-hospital death (OR and 95% CI: 4.12 and 2.04–8.33, *p*<0.0001). Age, the severity of baseline chest CT findings, number of comorbidities, total bilirubin, and lymphocyte counts were identified as risk factors for in-hospital death. The multivariate analyses revealed that a per point increase in the CONUT score was associated with an increased risk of in-hospital death in the adjusted model (OR and 95% CI: 1.44 and 1.03–2.01). On multivariate regression analysis, GNRI was independently associated with in-hospital mortality. Univariate analysis revealed that the OR for in-hospital death was 0.89 (95% CI 0.82–0.97, *p* < 0.0061). After adjusting for age, total bilirubin, numbers of comorbidity (diabetes, cardiovascular disease, chronic respiratory disease, chronic liver disease, and chronic kidney disease), lymphocyte counts, and chest CT findings, a logistic multivariate analysis found that the OR for in-hospital death was 0.89 (95% CI 0.79–0.998, *p* < 0.0458) for GNRI. GNRI ≤100 showed a 34.40-fold (*p* = 0.0142) increase in the incidences of in-hospital death compared with GNRI > 100 after adjusting for confounding factors (Table 7). To address the implication of PNI as an independent prognostic predictor in patients with COVID-19, 2 models of multivariate analysis were prepared. After adjustment, the PNI score in addition to age and CT findings predicted the in-hospital mortality (Table 8). Univariate analysis revealed that the OR of PNI for in-hospital death was 0.86 (95% CI 0.81–0.91, *p* < 0.0001). After adjusting for age, total bilirubin, numbers of comorbidity, and chest CT findings, the OR was 0.87 (95% CI 0.81–0.94, *p* = 0.0002) for PNI. PNI ≤ 44 showed a 7.58-fold (*p* = 0.0007) increase in the incidences of in-hospital death compared with PNI > 44 after adjusting for confounding factors. Consequently, the prognostic significance of PNI, CONUT score, and GNRI remained constant for both mortalities in nominal and continuous variables.

**Table 5:** The multivariate logistic regression analysis on the in-hospital mortality

	Model 1			Model 2		
	OR	95%CI	*p*-value	OR	95%CI	*p*-value
Chest CT findings	3.31	1.61–6.79	0.0011	3.72	1.86–7.43	0.0002
Numbers of comorbidity	1.30	0.74–2.30	0.3622	2.02	1.28–3.19	0.0025
Total bilirubin	1.05	0.97–1.14	0.2037	1.04	0.98–1.10	0.1919
Age	1.05	0.999–1.10	0.0575	1.04	1.003–1.08	0.0342
Total lymphocyte count	0.06	0.02–0.23	< 0.0001			
C-statistic	0.92			0.88		

Model 1 was adjusted by baseline the severity of chest computed tomography (CT) findings, numbers of comorbidities, total bilirubin, age, and total lymphocyte count. Model 2 was adjusted by baseline chest CT findings, numbers of comorbidities, total bilirubin, and age. *OR* Odds ratio, *CI* Confidence interval

**Table 6:** The association between controlling nutritional status (CONUT) score and in-hospital mortality

	Univariate model			Model 1			Model2		
	OR	95%CI	*p*-value	OR	95%CI	*p*-value	OR	95%CI	*p*-value
CONUT score	1.58	1.28–1.94	<0.0001	1.44	1.03–2.01	0.0351			
Undernutrition degree	4.12	2.04–8.33	<0.0001				2.48	0.86–7.15	0.0914
Chest CT findings				6.75	1.60–28.46	0.0093	7.16	1.70–30.16	0.0073
Numbers of comorbidity				1.43	0.63–3.25	0.3936	1.57	0.71–3.47	0.2667
Total bilirubin				1.14	1.0002–1.3	0.0496	1.14	1.01–1.30	0.0406
Age				1.09	1.001–1.18	0.0482	1.07	0.99–1.15	0.0790
C-statistic				0.95			0.94		

*OR* Odds ratio, *CI* Confidence interval, *CT* Computed tomography

**Table 7:** The association between geriatric nutritional risk index (GNRI) and in-hospital mortality

	Univariate model			Model 1			Model2		
	OR	95%CI	*p*-value	OR	95%CI	*p*-value	OR	95%CI	*p*-value
GNRI	0.89	0.82–0.97	0.0061				0.89	0.79–0.998	0.0458
GNRI≤100	14.82	1.72–127.79	0.0142	34.40	1.57–752.78	0.0246			
Chest CT findings				5.04	1.22–20.78	0.0251	3.88	1.23–12.21	0.0207
Total lymphocyte count				0.28	0.03–2.17	0.2211	0.25	0.03–2.28	0.2193
Numbers of comorbidity				1.77	0.64–4.93	0.2728	1.59	0.56–4.45	0.3814
Total bilirubin				1.15	1.02–1.31	0.0267	1.13	1.01–1.25	0.0279
Age				0.96	0.86–1.07	0.4422	0.98	0.89–1.08	0.6928
C-statistic				0.91			0.91		

*OR* Odds ratio, *CI* Confidence interval, *CT* Computed tomography

**Table 8:** The association between prognostic nutritional index (PNI) and in-hospital mortality

	Univariate model			Model 1			Model2		
	OR	95%CI	*p*-value	OR	95%CI	*p*-value	OR	95%CI	*p*-value
PNI	0.86	0.81–0.91	<0.0001	0.87	0.81–0.94	0.0002			
PNI ≤ 44	9.26	3.68–23.29	<0.0001				7.58	2.35–24.50	0.0007
Chest CT findings				3.58	1.72–7.43	0.0006	3.87	1.88–7.94	0.0002
Numbers of comorbidity				1.32	0.76–2.27	0.3236	1.43	0.85–2.41	0.1798
Total bilirubin				1.05	0.97–1.13	0.2028	1.06	0.99–1.14	0.0980
Age				1.05	1.003–1.10	0.0400	1.05	1.003–1.10	0.0348
C-statistic				0.91			0.90		

*OR* Odds ratio, *CI* Confidence interval, *CT* Computed tomography

## Discussion

The present study was the first to concern about the risk for malnutrition and provide evidence of nutritional risk screening in predicting outcomes for severe and critically ill patients infected with COVID-19. The present study showed that nutritional status, assessed using CONUT scores, GNRI, and PNI was significantly associated with poor in-hospital mortality in patients with COVID-19 in a multicenter setting, after adjusting for established risk factors. The results of this study suggested that the evaluation of risk for malnutrition was important for risk stratification.

The COVID-19 pandemic has spread throughout the world and has become a major public health threat. It is vital to offer optimal therapy to severe and critically ill patients and reduce the mortality caused by COVID-19. Recently, studies have shown that COVID-19 is largely dependent on certain socio-environmental factors, such as temperature [15–18], humidity [19–21], environmental pollution [22], and smoking [23], as well as clinical characteristics, such as comorbidity, laboratory test, symptoms, age, X-ray abnormality and functional status [13, 14]. To date, however, no studies have assessed the impact of objective nutritional parameters on COVID-19. Expert consensus on COVID-19 suggested that nutritional risk screening should be conducted among in-hospital COVID-19 patients [8, 24]. Given recent research gaps, the present study attempted to assess the impact of health status (i.e. nutritional status) on COVID-19 in China. The findings of the present study provided important evidence for recognizing patients at risk in addition to the established diagnostic criteria.

The nutritional status reflects the general condition of a patient, including physical condition, protein turnover, and immune-competence. Albumin, a component of the GNRI, CONUT score, and PNI, is the major protein in human plasma and the most abundant protein in the extracellular component [25]. Albumin synthesis is regulated by stimuli including nutrient intake, insulin levels, and oncotic pressure. Hypoalbuminemia is therefore thought to result from malnutrition, inflammation, or cachexia. Lymphocytopenia is considered to be related to physiological stress due to corticosteroid release and reflects a poorly regulated immune response [26].

The present study showed upward trends in inflammatory indexes along with the exacerbation of COVID-19, such as C-reactive protein and interleukin levels. Previous studies have theorized that inflammation might promote a generally catabolic state, stimulating protein degradation and the suppression of protein synthesis. Inflammation can also induce anorexia, aggravating the situation of malnutrition/undernutrition [27, 28]. Similarly, changes in metabolic indicators are also noteworthy. Reduced albumin, increased serum creatinine and blood urea nitrogen warn that severe and critically ill patients are at nutritional risk [7, 29]. Nevertheless, an increasing body of evidence suggests that it is a good marker for prognosis associated with malnutrition, and is even better for monitoring refeeding efficacy despite inflammation [30, 31]. However, it is worth noting that serum creatinine and blood urea nitrogen are more likely related to renal function especially in critically ill patients with complications in clinical practice. Therefore, composite indicators of nutritional status with high specificity are needed.

The risk for malnutrition among COVID-19 patients is caused by imbalances in energy intake and expenditure. Firstly, a high state of catabolism due to fever, over-activity of respiratory muscles, and the subsequent endocrine disorders result in the acceleration of gluconeogenesis, protein breakdown, and fat oxidation. Secondly, due to dyspnea mechanical ventilation and disturbance of consciousness, the patients may suffer from insufficient dietary intake. Thirdly, the direct attack of COVID-19 on the gastrointestinal tract results in nausea, diarrhea, and vomiting, with the prevalence of approximately 8.3%, 18.1 ~ 27.1%, and 4.0 ~ 5.9% [32, 33]. Finally, interventions such as mechanical ventilation and the use of antibiotics/antivirals cause hypoproteinemia and damage the digestive system [34–37].

There is no universally accepted definition of malnutrition or a gold-standard methodology for nutritional screening. In the present study, a per point increase in the CONUT score was associated with an increased risk of in-hospital death, as well as age, level of total bilirubin, and chest CT findings at admission. The present study indicated that nutritional screening using the CONUT score should be taken into consideration for COVID-19 patients. The CONUT score was first reported by Ignacio de Ulı’barri et al. as an objective screening tool for identifying undernutrition in a hospital population [9]. GNRI was first reported by Bouillanne et al. [11] in 2005 as a simple and accurate tool for predicting the risks of morbidity and mortality in hospitalized elderly patients. They defined 4 grades of nutrition-related risk: major risk (GNRI < 82), moderate risk (GNRI 82 to < 92), low risk (GNRI 92 to 98), and no risk (GNRI > 98). Many of the previous studies adopted GNRI cut-off points of 92 or 98. In the present study, the cut-off of GNRI was within the normal range, but the decrease of GNRI still predicted worse clinical outcomes after adjusting for important covariates. A possible explanation for this could be that patients in the present study had a better baseline clinical condition than patients in previous studies, and not only elderly or chronic kidney disease patients but also young or nonchronic kidney disease patients were included. It has been reported that GNRI is an independent prognostic factor for short-term in-hospital mortality in elderly patients with sepsis [38] since the 28-day mortality of very high-risk patients (GNRI < 82) has been increased sixfold [39]. Also, Wang et al. emphasized that GNRI should be of concern to clinicians as a potential prognostic predictor of COVID-19 based on the recently published preliminary results [40]. Given that GNRI was a simple, objective, and rapid method relating to patients’ nutritional status with short- and long-term outcomes, it should be considered as a potential predictor of COVID-19 severity and survival, regardless of patients’ comorbidities.

To date, several nutrition indicators have been reported, such as serum albumin, total cholesterol level, the Mini- Nutritional Assessment (MNA) [41], the Subjective Global Assessment (SGA) [42]. Assessments using a single indicator of malnutrition, such as serum albumin or total cholesterol level, may be affected by various factors and not provide adequate information. The MNA and SGA require subjective data evaluated by medical staff. A previous study demonstrated that inflammatory response reduced albumin synthesis [43]. A retrospective, observational study conducted by Zhao et al. showed that the most severe and critically ill patients infected with SARS-CoV-2 were at nutritional risk assessed by Nutritional Risk Screening 2002 (NRS) [44]. Frailty is one of the potential important junctions between poor nutritional status and worsened health outcomes. It is defined as a clinically identifiable state of increased physiologic vulnerability and dysfunction [45, 46]. Frailty is a measure of overall health and due to its relevance to immune function and risk of respiratory viral infection [47], could be also used as a significant prognostic factor for COVID-19. Besides, functional status could be a promising prognostic factor for patients suffering from COVID-19, as impaired physical function was independently associated with the worst outcomes in hospitalized patients with community-acquired pneumonia, according to a recent prospective study [48].

The aforementioned findings suggest that the incorporation of patients’ functional status measurement into patient assessment may improve the prognostic ability of current risk classification systems to predict mortality from COVID-19 pneumonia. The CONUT score includes serum albumin, total cholesterol levels, and total lymphocyte count for the assessment of nutritional status, while the PNI includes only albumin and lymphocyte count. GNRI is measured using both serum albumin and BMI. These three indexes, CONUT score, PNI, and GNRI, can be calculated using objective parameters and are originally developed to assess the nutritional status of patients with malignant diseases. The results of the discrimination analysis did not show significant improvement after adding the nutritional status. One possible reason was that the incident rate of in-hospital death was 8.5%. However, multivariate logistic regression analysis clearly showed that worse nutritional status was related to the higher risk of in-hospital mortality.

In the present study, the GNRI had a relatively high and significant OR for in-hospital mortality compared to the CONUT score and the PNI. These factors should be considered for risk stratification to detect high-risk individuals. However, further research should be carried out to elucidate this promising, time-saving method.

The present study had several limitations. First, this was a retrospective, observational study with a small sample size, and unknown confounders might influence the outcomes irrespective of the analytical adjustments. Besides, all of the patients were Chinese, so patients of different ethnicities/races needed to establish their reference. Second, the present study evaluated the risk for malnutrition once at admission and did not assess its changes. Meanwhile, only in-hospital mortality was analyzed. Thus, it was not possible to determine the long-term effects of nutrition risk. Third, the interaction between risk for malnutrition and the presence of the severity of COVID-19, which could affect especially the lymphocyte count, was not further explored. Finally, a validation study of the screening tool, however, was not examined. A prospective, long-term cohort study was needed to further verify the findings of the present study.

This was the first study to describe the risk for malnutrition in patients with COVID-19 using three nutritional indices. From the overall analysis, it was clear that nutritional status affected in-hospital mortality. The study was conducted in the hotspot cities in China. The results of this study are not only a major public health concern for these cities, but also for those that are likely to withstand a pandemic in the coming days during the current COVID-19 outbreak.

## Conclusion

Baseline nutritional risk, assessed by GNRI, PNI, or CONUT score, was a prognostic indicator for in-hospital mortality of COVID-19. The combination of these indicators with the traditional risk prediction model may provide a rapid and low-cost prognostic tool for COVID-19. Nutritional screening may be a positive strategy during this pandemic, and meanwhile, further studies should be performed to establish nutritional intervention strategies or long-term outcomes of the infection.

## Data Availability

All data generated or analyzed during this study are included in this published article.
